# Association of Circulating 25(OH)D and Lower Urinary Tract Symptoms: A Four-Year Prospective Study among Elderly Chinese Men

**DOI:** 10.3390/nu8050273

**Published:** 2016-05-07

**Authors:** Zhao-Min Liu, Carmen Ka Man Wong, Dicken Chan, Jean Woo, Yu-Ming Chen, Bailing Chen, Lap-Ah Tse, Samuel Yeung-Shan Wong

**Affiliations:** 1 Division of Family Medicine and Primary Care, Jockey Club School of Public Health and Primary Care, the Chinese University of Hong Kong, Hong Kong 99977, China; liuzhaomin@cuhk.edu.hk (Z.-M.L.); carmenwong@cuhk.edu.hk (C.K.M.W.); dicken@cuhk.edu.hk (D.C.); 2 Department of Medicine and Therapeutics, the Chinese University of Hong Kong, Hong Kong 99977, China; jeanwoowong@cuhk.edu.hk; 3 Department of Medical Statistics and Epidemiology, School of Public Health, Sun Yat-sen University, Guangzhou 51000, China; chenyum@mail.sysu.edu.cn; 4 Department of Spine Surgery, the First Affiliated Hospital of Sun Yat-sen University, Guangzhou 51000, China; chenbl2012@163.com; 5 Division of Occupational and Environmental Health, Jockey Club School of Public Health and Primary Care, the Chinese University of Hong Kong, Hong Kong 99977, China; shelly@cuhk.edu.hk

**Keywords:** lower urinary tract symptoms, elderly men, serum 25(OH)D, international prostate symptoms score

## Abstract

The role of vitamin D in relation to lower urinary tract symptoms (LUTS) remains inconclusive. This four-year longitudinal study aims to explore the association of circulating 25(OH)D and LUTS in elderly Chinese men. Two thousand Chinese men aged 65 and older were recruited from a local community, of which 1998 (99.9%) at baseline and 1564 (78.2%) at four-year follow-up reported data on LUTS, and 988 of the randomly chosen subpopulation were assayed for serum 25(OH)D by radioimmunoassay at baseline. LUTS were evaluated by a validated International Prostate Symptoms Scale (IPSS). Data on demographic characteristics, lifestyle factors, health, and medications were collected. Serum parathyroid and sex steroid hormones and genotypes of vitamin D receptors were assayed. The association of serum 25(OH)D and LUTS was examined by using multivariable regression models. Serum 25(OH)D was not significantly associated with the changes of IPSS or the risk of LUTS in overall participants. However, among men with 25(OH)D ≤ 60 nmol/L, each 10 nmol/L increase of 25(OH)D over 0 nmol/L was significantly associated with 1.3 lower points of IPSS or a 51.6% decreased risk for moderate/severe LUTS four years later. Adjustment for serum androstenedione (*p* = 0.019) and dehydropiandrosterone (*p* = 0.037) attenuated the associations. Our study suggested that among individuals with low vitamin D status, the increase of the 25(OH)D level may be associated with a lowered risk of LUTS.

## 1. Introduction

Lower urinary tract symptoms (LUTS), a cluster of chronic urinary symptoms in the bladder, prostate or urethra, represent one of the most common clinical complaints in elderly men [1]. LUTS result from a complex interplay of pathophysiologic features, comprising a progressive, age-related combination of storage, voiding, and post-micturition symptoms [2]. Men with overactive bladder symptoms (bladder-storage dysfunction) were found to have a high prevalence of LUTS and greater LUTS severity. Both urological and non-urological conditions may contribute to LUTS with benign prostatic hyperplasia (BPH) being one of the principal underlying causes of LUTS [3]. A recent survey in China reported a high prevalence with 78.8% of men above 40 years of age having at least one LUTS [4]. The pervasiveness of LUTS, along with its associated medical costs, requires further understanding of the causes of LUTS and the identification of modifiable risk factors in order to prevent or delay the symptom progression.

There is increasing evidence that vitamin D deficiency or insufficiency can be an important risk factor for various adverse health outcomes [5]. However, evidence of its role in relation to LUTS is not adequately characterized. *In vitro* and animal studies show that vitamin D reduces cellular proliferation and differentiation of prostate acting through vitamin D receptor (VDR) [6]. VDR polymorphisms may be involved in BPH pathogenesis by encoding growth factors [7]. The circulating hormones (*i.e*., parathyroid and sex hormones) may influence the biological actions of vitamin D on prostate [6,8]. Cross-sectional studies also suggest a vitamin D deficiency (25(OH)D less than 50 nmol/L) or an insufficiency (50–74.9 nmol/L) or a lack of dietary vitamin D intake was associated with the increased risk of LUTS or BPH [9,10,11]. A cohort study by Kristal *et al.* reported that an increased intake of vitamin D from both diet and supplements or just from supplements alone may have an impact on reducing BPH prevalence [12].

Despite a dietary intake of vitamin D, skin synthesis from sunlight exposure is the major source of vitamin D (80%–90%) in humans, and the circulating 25(OH)D is the optimal indicator of overall vitamin D status [13]. To the best of our knowledge, no biomarker-based longitudinal studies have examined the relationship of serum vitamin D and the risk of LUTS and no study has explored the temporal relationship among Asian populations. Our analysis used data from a prospective study in elderly Chinese men to examine the association of serum 25(OH)D and LUTS, and we also explored the potential mechanisms on VDR gene polymorphisms and sex hormones.

## 2. Materials and Methods

### 2.1. Participants

This was a four-year longitudinal study (Mr. OS Hong Kong) among 2000 Chinese men aged 65 and older. The details of the subjects’ recruitment have been described previously [14]. In brief, participants were recruited from the local community from 2001 to 2003 by placing recruitment notices in housing estates and community centers. The inclusion criteria required all participants to be able to walk or take public transport to the study site at the University’s teaching hospital in Shatin. Stratified sampling was adopted to have approximately 33% of subjects in each of the following age groups: 65–69, 70–74, and ≥75 years. Subjects were recruited on a voluntary basis, and recruitment was terminated once the target sample size was reached. Subjects were invited to the research center for interviews and physical examination. The study protocol was approved by The Chinese University of Hong Kong Ethics Committee, and informed consent was obtained from all subjects before enrollment.

### 2.2. Data Collection

Subjects were interviewed by using a set of structured and standardized questionnaires or an examination form covering the following aspects: demographic characteristics; health and medication; and lifestyle factors (such as cigarette smoking, alcohol consumption, dietary intake, and physical activity). Blood was taken after an overnight fast from all subjects. EDTA whole blood and serum samples were kept frozen at −80 °C.

#### 2.2.1. Lower Urinary Tract Symptoms (LUTS)

The presence and severity of LUTS were assessed by a validated Chinese version of the International Prostatic Symptoms Score (IPSS) [15,16]; the data for which were collected at baseline and at the end of two-year and four-year follow-ups. The IPSS is an eight-item questionnaire that includes seven symptom questions (*i.e*., nocturia, frequency, urgency, intermittency, weak stream, incomplete emptying, and straining) and one global quality-of-life question. For the seven symptom questions, each item refers to during the last month and each involves the assignment of a score from 1 to 5 for a total of 35 points. According to the IPSS, men were defined as severe LUTS if they scored ≥20; moderate LUTS if they scored 8–19 and mild LUTS with a score ≤7.

#### 2.2.2. Serum Analyses of 25(OH)D, Parathyroid Hormone (PTH), and Sex Hormones

A subpopulation of 988 men was randomly chosen by simple random sampling and had fasting venous sampling at baseline for assay of serum 25(OH)D and PTH. Serum was stored at −80 °C, and levels of 25(OH)D were measured by a competitive radioimmunoassay (DiaSorin, Stillwater, MN, USA). This assay measures both 25(OH)D_3_ and 25(OH)D_2_. Intra- and interassay coefficients of variations (CVs) were 6% and 18%, respectively. Serum intact PTH levels were measured by using an immune-luminometric assay (Diagnostic Products Corp., Los Angeles, CA, USA). The intra- and interassay CVs were 5% and 9%, respectively.

A total of 1489 subjects were randomly selected by simple random sampling to have stored serum analyzed for precursors-dehydroepiandrosterone (DHEA), dehydroepiandrosterone sulfate, 5-androstene-3b,17b-diol (5-Diol), and androstenedione (4-Dione), among which 399 subjects were randomly selected for testing of sex hormone binding globulin (SHBG), testosterone, and estradiol. Free fractions of testosterone and estradiol were calculated, as described by Sodergard [17]. All hormone assays were performed by Gas chromatography-mass spectrometry (GC/MS) [18].

#### 2.2.3. Genotyping of VDR Tag SNPs and the Major Haplotypes

Blood samples for DNA analyses were collected from 1680 individuals. DNA was extracted from whole blood by using standard phenol/chloroform extraction [19]. Genotypes of VDR were determined by polymerase chain reaction (PCR) amplification. The SNPs (rs731236 and rs9729) genotyped in the study cohorts and used as tag SNPs were all in Hardy-Weinberg equilibrium, and they were used for prediction of the major haplotypes in the study populations.

#### 2.2.4. Other Covariates

Cigarette smoking and alcohol consumption were investigated based on self-report by using validated methods. Anthropometric measures were conducted at the baseline, two-year and four-year follow-ups by a standardized protocol. Body weight was measured with subjects wearing a light gown by the Physician Balance Beam Scale (Healthometer, Alsip, IL, USA). Height was measured by the Holtain Harpenden stadiometer (Holtain Ltd., Crosswell, UK). Body mass index (BMI) was calculated as: body weight (kg)/m^2^. Physical activity was assessed by using the Physical Activity Scale of the Elderly (PASE) [20]. Dietary intake was assessed at baseline by using a validated semi-quantitative food frequency questionnaire (FFQ) [21].

### 2.3. Statistical Analysis

Statistical analyses were performed by using the statistical package SPSS version 19.0 (SPSS Inc., Chicago, IL, USA). Α level of 5% was used as the level of significance. Data were checked for normality, and transformation was made whenever appropriate (serum 25(OH)D, and IPSS were Log_10_ transformed in a linear regression model). Baseline characteristics were compared by independent *t*-test for continuous variables or by a chi-square test for categorical variables between participants with mild (IPSS < 8) and moderate/severe (IPSS ≥ 8) LUTS, and between participants with and without testing of 25(OH)D.

Multivariate linear regression models were applied to examine the associations of serum 25(OH)D level with the IPSS changes from baseline at two-year and four-year follow-ups by adjustment for potential covariates. Variables found to be associated with LUTS in univariate analyses were put into multiple regression models. Adjusted variables were age, seasons, educational level, baseline IPSS, cigarette smoking (no, current, ever), coffee (mL/day), alcohol (g/day), calcium supplement (yes or no), prostate medication (yes or no), BMI, history of fracture, hypertension, stroke, diabetes (yes or no), PASE total score, dietary energy (kcal/day), and isoflavones (mg/day) intakes. To avoid reverse causation, sensitivity analysis by the exclusion of subjects with severe LUTS was conducted (*n* = 904). Stratification analyses by different seasons (winter/spring *vs.* autumn/summer) and 25(OH)D levels (≤60 *vs.* >60 nmol/L) were also conducted to examine whether the associations of 25(OH)D and IPSS changes were different. The cut-off of 25(OH)D level at 60 nmol/L was suggested by the General Linear Model, in which the only significant difference was observed between quintile 1 (≤60 nmol/L) and quintile 2 (60–80 nmol/L). The cut-point at 60 nmol/L gave the best discrimination between those with and without an improvement in LUTS, and this was further confirmed by multivariable fractional polynomial models. We also used the conventional cut-points of 25(OH)D at 50 or 75 nmol/L, which are recommended by the Institute of Medicine [22], to repeat the data analysis. However, only marginally inverse associations were observed between 25(OH)D and IPSS changes. Thus, the stratified analyses were conducted among participants with 25(OH)D ≤ 60 and >60 nmol/L.

Binary logistic regression was performed to further characterize the relationship between the serum 25(OH)D levels and the risk of moderate/severe LUTS at four-year follow-up after controlling for the above confounders. Subjects with severe LUTS (IPSS ≥ 20) at baseline were excluded from this analysis (*n* = 154, 7.7%). To explore the influence of season on this association, the interaction term of 25(OH)D and seasons was included in the regression model. To investigate the possible mechanisms related to VDR polymorphisms, we conducted exploratory analyses to test the interactions of genetic factors (SNPs and VDR haplotypes) and 25(OH)D with respect to IPSS by adding a cross-product term to the models, and we assessed significance with the Wald test. We also adjusted serum hormones in the regression models between 25(OH)D and absolute IPSS changes at four-year follow-up to examine whether the hormones mediated these associations.

## 3. Results

### 3.1. Baseline Characteristics of Participants with Mild and Moderate/Severe LUTS

Among the 2000 recruited elderly men, 1998 (99.9%) at baseline, 1744 (87.2%) at two-year and 1564 (78.2%) at four-year follow-up reported data on LUTS. After excluding those with a history of bladder cancer (*n* = 13) and prostate cancer (*n* = 16), analyses were performed among 1971 elderly men at baseline. The baseline characteristics of overall participants are presented in Table 1. Compared with those with mild LUTS, the men with moderate/severe LUTS were older, less educated, more likely to be under antihypertensive treatment, had lower dietary energy, protein and calcium intakes, less alcohol and coffee drinking, lower levels of physical activity, and lower serum bioavailable estradiol levels.

### 3.2. Baseline Characteristics of Participants with and without 25(OH)D Testing

Comparisons of baseline characteristics between those with and without 25(OH)D testing are presented in Table 2. There were no significant differences in educational level, smoking status, alcohol use, and dietary factors between the men with and without 25(OH)D measurement. However, the men with 25(OH)D measurement at baseline were older, engaged in more physical activity, had a lower BMI and higher baseline IPSS (*p* < 0.05).

### 3.3. The Associations of Serum 25(OH)D with IPSS Changes and Risk of Moderate/Severe LUTS

After adjustment for potential confounders, longitudinal analyses in overall participants showed that serum 25(OH)D level in continuous form was not significantly associated with the IPSS changes from baseline (Table 3), or with the risk of moderate/severe LUTS (Table 4) at two-year and four-year follow-ups in both the crude or adjusted models. Sensitivity analysis by the exclusion of participants with severe LUTS and stratified analysis by seasons indicated similar non-significant results with those of overall participants.

However, subgroup analysis among the men with 25(OH)D level ≤ 60 nmol/L indicated that there was a significant association of serum 25(OH)D and decreased scores of LUTS (unadjusted coefficient B = −0.155, *p* = 0.02), or a reduced risk of moderate/severe LUTS with a risk ratio of 0.930 (95% CI: 0.872, 0.992, *p* = 0.02) at four-year follow-up after adjusting for covariates (Table 3 and Table 4). Analysis among the subclasses of LUTS suggested that the major improvement in LUTS could be occurring in urinary intermittency, frequency, and urgency (Table 3). There was no significant interaction between 25(OH)D and seasons (*p* = 0.172). In addition, no significant association (either linear or non-linear) was detected among those with a 25(OH)D level > 60 nmol/L.

Among men with 25(OH)D ≤ 60 nmol/L, there were no significant interactions between their serum 25(OH)D level and their genetic factors of VDR (SNPs and VDR haplotypes) that influenced the risk of moderate/severe LUTS. The *p* values for the interactions were 0.580, 0.525, 0.580, 0.859, and 0.947 for DNArs9729, DNArs731236, Hap1, Hap2, and Hap3, respectively. Adjustment for serum adrostenedione (*p* = 0.019) and dehydropiandrosterone (*p* = 0.037) levels significantly attenuated the association of 25(OH)D and the four-year change of IPSS in the univariate regression models (Table S1).

## 4. Discussion

To the best of our knowledge, this is the first prospective study that examined the temporal association of serum vitamin D and LUTS in community-dwelling elderly Chinese men. In this four-year cohort study, serum 25(OH)D was not significantly associated with the severity of LUTS in overall participants. However, a significant association between the increase of 25(OH)D and the decreased risk of LUTS was observed among men with a low 25(OH)D status (≤60 nmol/L). For individuals with 25(OH)D ≤ 60 nmol/L, each 10 nmol/L increase of 25(OH)D over 0 nmol/L was significantly associated with 1.3 lower points of IPSS or a 51.6% decreased risk for moderate or severe LUTS. Although the effect size was modest relative to the medication treatment [23], it may still have important public health implications for the improvement of LUTS in consideration of the high prevalence of LUTS or BPH in elderly men, especially if a possible intervention offers high compliance through a well-tolerated, inexpensive, and safe supplement such as vitamin D.

### 4.1. Results Explanation

Our findings among elderly men with low vitamin D status are consistent with previous cross-sectional studies in men [9,10] and women [24], which show that low serum vitamin D was associated with an increased odds of LUTS. The cross-sectional data among US adults [9] reported that vitamin D deficiency (25(OH)D < 50 nmol/L) was associated with the presence of moderate-severe urinary incontinence (OR 1.8, 95% CI: 1.1–3.0) and at least one LUTS (OR 1.4, 95% CI: 1.0–2.0). Kristal *et al.* [12] investigated the dietary factors and the seven-year incidence of symptomatic BPH among men aged 54–86, and they reported that the highest quintile of total vitamin D intake was associated with an 18% reduced risk of BPH than the lowest one. However, total vitamin D (diet plus supplement) intake in this study was self-reported and circulating vitamin D status was unknown. A randomized controlled trial [25] suggested a promising result with vitamin D supplementation on BPH. The study indicated that vitamin D analogue decreased 2.9% prostate volume in BPH patients; however, no significant changes were found in scores of urological symptoms. Thus, further interventional studies among men with low vitamin D status are needed to clarify the effect of vitamin D on LUTS or BPH.

In this four-year longitudinal study, no significant association was observed between the serum 25(OH)D level and the changes of IPSS or the risk of LUTS in the overall population. The lack of association may be due to the relatively short duration (four years), or the relatively adequate vitamin D levels in our study. However, prospective studies [26,27,28] with shorter duration than ours still observed significant associations of lifestyle factors with the progression of LUTS. Although the typical diet in the Chinese population is limited in vitamin-D-rich or fortified foods, the higher functional status of the participants and also their likely greater sun exposure from outdoor activities than in the general population of a similar age may account for the relatively higher vitamin D status in our study [29]. Vitamin D deficiency may also suggest an overall frail condition represented by a low vitamin D level; however, the adjustment for multiple comorbid factors (diabetes, hypertension stroke, hypertension, cancers, *etc*.) did not change the significance of the findings.

Previous observations suggested that there are significant associations between serum 25(OH)D and health parameters, and these were mostly reported in populations with lower vitamin D status [30,31,32]. The thresholds of 25(OH)D levels have varied with different health outcomes, settings, study designs, and the analytical methods used to measure 25(OH)D [33]. A study in the Netherlands reported that the threshold of about 40 nmol/L existed for bone turnover markers, 50 nmol/L for bone mineral density, and 60 nmol/L for physical performance [34]. Another case-control study [35] showed a significant non-linear relationship that 25(OH)D was inversely associated with hip fracture only below 70 nmol/L. Different thresholds of 25(OH)D insufficiency for various health outcomes may be explained by the extra-renal hydroxylation of 25(OH)D to the active metabolite 1,25(OH)_2_D in different organs because 1,25(OH)_2_D is capable of acting via autocrine and paracrine mechanisms [33]. Our results suggested that the threshold of 25(OH)D corresponding to improved LUTS might be at 60 nmol/L. However, the findings were derived from a small subpopulation (*n* = 196) and based on exploratory analysis in nature; therefore, future large prospective studies are necessary to confirm the threshold of 25(OH)D on the improvement of prostate symptoms.

Our longitudinal data reported an inverse association between 25(OH)D and the decreased risk of LUTS among those with low 25(OH)D status. This association may not be related to VDR gene polymorphisms, but it may be mediated by serum adrostenedione or dehydropiandrosterone levels. *In vitro* and animal studies have indicated that vitamin D can regulate the gene expression and enzyme activity of a number of steroidogenic enzymes, and decrease the production of corticosterone, androstenedione, and dehydroepiandrosterone [36], which could thus moderate the hormonally regulated prostate growth and smooth muscle tone that cause BPH and LUTS [37]. Our findings are consistent with preclinical trials that vitamin D could decrease prostate cell proliferation and that the effect was induced by sex hormones or growth-promoting molecules [8].

Studies of animal models and human cell lines have shown low vitamin D status could promote cell proliferation and decrease apoptosis in normal prostate cells [38]. In addition, VDR agonist could not only inhibit prostate growth and inflammation [39], but also modulate bladder contractile mechanisms [40] and thereby prevent bladder outlet obstruction [41] *in vitro* and *in vivo*, which provides plausible mechanisms on vitamin D and a reduced risk of LUTS. VDR are known to exist in prostate and bladder tissues and the prostate has been recognized as an extra-renal site for the synthesis of 1,25(OH)_2_D_3_. Evidence suggests that VDR polymorphisms are important in modifying the receptor function [42]. However, our findings are in line with recent meta-analysis that VDR gene polymorphisms are not associated with the risk of BPH either for Caucasians or Asians [43].

### 4.2. Strengths

There are several strengths to our study. Firstly, we employed a prospective design and included a large number of potential confounding factors. Secondly, we tested the serum 25(OH)D level which best reflects overall vitamin D status, thus providing the most direct evidence on the association of vitamin D and LUTS. Thirdly, the possibility of reverse causality appears unlikely because the association of vitamin D and LUTS or prostate health is not extensively acknowledged and there is little concern that men with moderate or severe LUTS will seek vitamin D supplementation. In addition, sensitivity analysis with the exclusion of participants with severe LUTS reported similar findings with overall participants.

### 4.3. Limitations

Our study has several limitations. Firstly, serum 25(OH)D data were available only in half of the male subjects. In addition, 25(OH)D levels were measured at a single point of time and this may not adequately reflect long-term vitamin D status. Nevertheless, previous reports suggested a strong correlation between 25(OH)D values measured at three years (*r* = 0.7) [44] and 11 years (*r* = 0.5) [45] apart.

Secondly, the significant inverse association of 25(OH)D and IPSS change among men with low vitamin D levels was derived from a small subgroup analysis, and therefore the findings should be treated with caution. Further prospective studies with larger numbers of participants or studies specifically designed among men with low vitamin D status are needed to confirm this finding.

Thirdly, LUTS in our study was evaluated by using a validated scale. The IPSS has many advantages because it is self-administered, sensitive to changes, and generalizable to different populations and socio-economic groups. However, the IPSS does not address urinary incontinence. It may underestimate LUTS severity in men with storage symptoms. Additionally, an assessment of surgical BPH or diagnosed BPH was not conducted. However, BPH is a significant contributor to LUTS in men. The diagnostic instruments for BPH would be more time-consuming and invasive and impractical in a large cohort like ours. Moreover, LUTS have been shown to exhibit progression and regression over time. Thus, the one-month recall timeframe of the IPSS questions may confound our results. However, the exclusion of severe LUTS at baseline and the measurement of IPSS changes over time could reduce the impact of these fluctuations in our study.

Finally, the study sample consisted of volunteers who were all of a higher educational level and more likely to be married compared with the general Hong Kong population in the same sex and age groups [46], and therefore the results may not be entirely generalizable. However, the selection bias would not affect the estimates of the exposure–outcome associations [47].

## 5. Conclusions

This four-year cohort study among elderly Chinese men indicated that, although the serum 25(OH)D level was not associated with LUTS in the overall population, among those with a low vitamin D (≤60 nmol/L) status, 25(OH)D may be associated with a lowered risk of LUTS. Further prospective studies specifically conducted among elderly men with low vitamin D status are warranted to confirm this finding.

## Figures and Tables

**Table 1:** Participants’ characteristics at baseline.

	Overall Participants	IPSS < 8 (Mild)	IPSS ≥ 8 (Moderate/Severe)	*p*
*n*	1998	1178	820	
Age (year)	72.4 ± 5.0	72.0 ± 4.8	73.0 ± 5.2	0.006
Body mass index (BMI, kg/m^2^)	23.4 ± 3.1	23.4 ± 3.0	23.4 ± 3.3	0.99
Education (university or above, %)	271 (13.6)	190 (16.1)	81 (9.9)	0.001
Marriage (married or cohabiting, %)	1758 (88.0)	1039 (88.2)	719 (87.7)	0.73
Smoking (%)				0.17
No smoking	724 (36.2)	445 (37.8)	279 (34.0)	
Current smoking	1036 (51.9)	591 (50.2)	445 (54.3)	
Passive smoking	238 (11.9)	142 (12.1)	96 (11.7)	
Calcium supplement (%)	193 (9.7)	112 (9.5)	81 (9.9)	0.78
Multi-vitamin use (%)	91 (4.6)	60 (5.1)	31 (3.8)	0.17
Antihypertensive medication (%)	897 (44.9)	464 (39.4)	433 (52.8)	0.001
Prostate medication (%)	305 (15.3)	115 (9.8)	190 (23.2)	<0.001
Medical history (%)				
Fracture	274 (13.7)	167 (14.2)	107 (13.0)	0.47
Diabetes	293 (14.7)	174 (14.8)	119 (14.5)	0.87
Stroke	109 (5.5)	59 (5.0)	50 (6.1)	0.29
Hypertension	835 (41.8)	466 (39.6)	369 (45.0)	0.02
Bladder cancer	13 (0.6)	6 (0.5)	7 (0.9)	0.35
Prostate cancer	16 (0.8)	6 (0.5)	10 (1.2)	0.08
Dietary factors				
Energy (Kcal/day)	2099.5 ± 586.5	2126.8 ± 591.7	2061.1 ± 577.6	0.01
Protein (g/day)	87.7 ± 35.3	89.0 ± 35.3	85.7 ± 35.2	0.04
Fat (g/day)	67.7 ± 24.5	68.2 ± 24.8	67.0 ± 24.0	0.28
Calcium (mg/day)	628.3 ± 297.6	649.7 ± 300.0	597.6 ± 291.8	<0.001
Vitamin D (IU/day)	14.5 ± 25.4	14.7 ± 25.2	14.4 ± 25.7	0.79
Alcohol intake (g/day)	25.9 ± 113.7	31.8 ± 137.0	17.4 ± 66.4	0.01
Coffee drinking (mL/day)	30.5 ± 78.0	34.7 ± 83.8	24.5 ± 68.5	0.004
PASE total score		101.5 ± 52.3	91.3 ± 46.7	<0.001
Major VDR haplotype groups				
*n*	1606	948	658	
Homozygous Hap1 (%)	797 (49.6)	471 (49.7%)	326 (49.5%)	0.98
Homozygous Hap2 (%)	106 (6.6)	56 (5.9%)	50 (7.6%)	0.21
Homozygous Hap3 (%)	8 (0.3)	3 (0.3%)	5 (0.3%)	0.22
Heterozygous Hap1/Hap2 (%)	535 (33.3)	307 (32.4%)	228 (34.7%)	0.50
Heterozygous Hap1/Hap3 (%)	119 (7.4)	83 (8.8%)	36 (5.5%)	0.02
Heterozygous Hap2/Hap3 (%)	0	0	0	-
Serum hormones				
*n*	400	256	144	
SHBG (nmol/L)	42.9 ± 16.5	42.9 ± 16.3	42.9 ± 17.1	0.99
Bioavailable estradiol (pmol/L)	77.7 ± 21.3	79.4 ± 21.5	74.7 ± 20.8	0.04
Total testerosterone (nmol/L)	17.8 ± 6.5	18.1 ± 6.5	17.2 ± 6.6	0.18
Free testosterone (nmol/L)	0.316 ± 0.119	0.323 ± 0.121	0.304 ± 0.114	0.11
*n* *	1487	860	627	
Androstenedione (ng/mL)	0.731 ± 0.236	0.733 ± 0.233	0.728 ± 0.239	0.65
Dehydroepiandrosterone sulfate (ng/mL)	1.89 ± 1.00	1.92 ± 0.99	1.84 ± 1.00	0.13
5-androstene-3b,17b-diol (ng/mL)	0.68 ± 0.35	0.68 ± 0.35	0.67 ± 0.36	0.30
*n*	988	546	442	
25(OH)D (nmol/L)	78.3 ± 21.4	78.5 ± 21.1	78.0 ± 21.7	0.71
PTH (pmol/L)	4.49 ± 2.38	4.38 ± 2.09	4.63 ± 2.69	0.11

Data were presented as mean ± SD for continuous variables or *n* (%) for categorical variables. Lower Urinary Tract symptoms were evaluated by the International Prostate Symptom Score (IPSS); SHBG: Sex hormone-binding globulin. PASE: Physical Activity Scale for the Elderly; PTH: Parathyroid hormone. Body mass index (BMI) was calculated as: body weight (kg)/height·m^2^. Free fractions of testosterone and estradiol were calculated, as described by Sodergard. * Two men lacked data on IPSS, and 1487 subjects were analyzed.

**Table 2:** Participants’ characteristics by with and without testing of serum 25(OH)D at baseline.

	Testing for Serum 25(OH)D		*p*
	No	Yes	
*n*	1012	988	
Age (year)	72.0 ± 4.9	72.8 ± 5.0	0.001
Body mass index (BMI, kg/m^2^)	23.7 ± 3.1	23.2 ± 3.2	<0.001
Education (university or above, %)	134 (13.2)	137 (13.9)	0.10
Marriage (married or cohabiting, %)	895 (88.5)	864 (87.4)	0.19
Current smoking (%)	526 (52.0)	512 (51.8)	0.99
Multi-vitamin use (%)	44 (4.3)	47 (4.8)	0.66
Medications (%)			
Prostate medication	129 (12.7)	176 (17.8)	0.002
Anti-androgen use	3 (0.3)	4 (0.4)	0.68
Medical history (%)			
Fracture	135 (13.3)	139 (14.1)	0.67
Diabetes	136 (13.4)	157 (15.9)	0.12
Stroke	50 (4.9)	59 (6.0)	0.31
Hypertension	426 (42.1)	410 (41.5)	0.78
Heart diseases	98 (9.7)	103 (10.4)	0.58
PASE total score	94.7 ± 47.2	99.9 ± 53.1	0.02
Dietary factors			
Total energy (kcal/day)	2115 ± 603	2,083 ± 570	0.22
Protein (g/1000 Kcal/day)	41.1 ± 9.4	41.3 ± 9.5	0.77
Fat (g/1000 Kcal/day)	32.3 ± 7.3	32.2 ± 6.9	0.81
Alcohol intake (g/day)	28.2 ± 138.5	23.5 ± 80.7	0.35
Vitamin D (IU/day)	14.9 ± 29.9	14.2 ± 19.8	0.56
Total isoflavones (mg/day)	16.5 ± 26.9	14.8 ± 18.4	0.11
Baseline IPSS	7.1 ± 6.5	8.5 ± 7.2	<0.001

Data were presented as mean ± SD for continuous variables or *n* (%) for categorical variables. Lower Urinary Tract symptoms were evaluated by the International Prostate Symptom Score (IPSS); PASE: Physical Activity Scale for the Elderly; Body mass index (BMI) was calculated as: body weight (kg)/height·m^2^.

**Table 3:** Univariate and multivariate linear regression analyses on the association of serum 25(OH)D and the changes of International Prostate Symptom Score (IPPS) at two-year and four-year follow-ups as well as stratified analyses by different 25(OH)D levels, seasons and severity of LUTS.

	Crude		Adjusted	
	β	*p*	β	*p*
**All participants (*n* = 967)**				
IPSS change at 2-year FU	−0.002	0.93	−0.007	0.73
IPSS change at 4-year FU	−0.012	0.61	−0.005	0.80
**Stratified analysis by serum 25(OH)D level (> or ≤60 nmol/L)**				
**25(OH)D > 60 nmol/L (*n* = 771)**				
IPSS change at 2-year FU	−0.016	0.66	−0.006	0.86
IPSS change at 4-year FU	−0.046	0.20	−0.040	0.23
**25(OH)D ≤ 60 nmol/L (*n* = 196)**				
IPSS change at 2-year FU	−0.056	0.44	−0.596	0.55
IPSS change at 4-year FU	−0.155	0.02	−0.149	0.03
IPSS change in individual symptoms at 4-year FU				
Intermittency	−0.174	0.02	−0.145	0.05
Frequency	−0.121	0.09	−0.121	0.10
Incomplete emptying	−0.016	0.83	−0.023	0.75
Urgency	−0.128	0.07	−0.125	0.08
Slow/weak stream	−0.004	0.96	0.010	0.89
Straining to void	−0.002	0.98	−0.026	0.72
Nocturia	−0.081	0.26	−0.096	0.19
Quality of life	0.020	0.78	0.027	0.73
**Stratified analysis by seasons**				
**25(OH)D assessed in winter or spring (*n* = 466)**				
IPSS change at 2-year FU	0.001	0.99	0.001	0.99
IPSS change at 4-year FU	0.094	0.37	0.037	0.72
**25(OH)D assessed in summer or autumn (*n* = 501)**				
IPSS change at 2-year FU	0.023	0.76	0.011	0.88
IPSS change at 4-year FU	−0.015	0.85	−0.024	0.75
**Sensitivity analysis with exclusion of participants with severe LUTS (*n* = 904)**				
IPSS change at 2-year FU	0.006	0.93	−0.017	0.79
IPSS change at 4-year FU	−0.029	0.64	−0.031	0.62

Data analyses were conducted by univariate and multivariate linear regression models with IPSS changes as continuous variable; β: standardized B coefficient. The adjusted variables included age, season, educational level, baseline Prostate Symptom Score, cigarette smoking (no, current, ever), coffee(mL/day), alcohol (g/day), calcium supplement (yes or no), prostate medication (yes or no), body mass index (BMI), history of fracture, hypertension, stroke or diabetes (yes or no), PASE total score, dietary energy (kcal/day) and dietary isoflavones (mg/day) intake, *etc.* IPSS: International Prostate Symptoms Score; FU: follow-up; β: standardized B coefficient; PASE: Physical Activity Scale for the Elderly.

**Table 4:** Risk ratio (RR) and 95% CI confidence interval for moderate/severe Lower Urinary Tract Symptoms (LUTS) at two-year and four-year follow-ups according to the baseline serum 25(OH)D levels.

	No.	Univariate RR (95% CI)	*p*	Multivariate RR (95% CI)	*p*
All participants					
2-year FU	871	0.998 (0.991,1.004)	0.53	0.997 (0.990,1.004)	0.37
4-year FU	683	1.001(0.994,1.008)	0.79	1.000 (0.992,1.008)	0.98
Participants with 25(OH)D ≤ 60 nmol/L					
2-year FU	176	0.975(0.931, 1.021)	0.27	0.960 (0.911, 1.012)	0.13
4-year FU	139	0.937(0.888, 0.990)	0.02	0.930 (0.872,0.992)	0.02

Data were analyzed by binary logistic regression models with exclusion of men with severe lower urinary tract symptoms (PSS ≥ 20); RR: risk ratio; 95% CI: 95% confidence interval; FU: follow up; The adjusted variables include age, season, educational level, baseline Prostate Symptom Score, cigarette smoking (no, current, ever), coffee(mL/day), alcohol (g/day), calcium supplement (yes or no), prostate medication (yes or no), body mass index (BMI), history of fracture, hypertension, stroke, diabetes (yes or no), PASE total score, dietary energy (kcal/day), vitamin D (IU/day) and isoflavones (mg/day) intake *etc*. LUTS: Lower Urinary Tract Symptoms; PASE: Physical Activity Scale for the Elderly.

## Supplementary Materials

The following are available online at http://www.mdpi.com/2072-6643/8/5/273/s1. Table S1: Results of univariate analysis between serum 25(OH)D level and International Prostate Symptoms Score (IPSS) change at four-year follow-up by adjusting serum hormone levels in elderly men with baseline 25(OH)D ≤ 60 nmol/L (*n* = 196).

## Abbreviations

The following abbreviations are used in this manuscript:

LUTS	lower urinary tract symptoms
IPSS	International Prostate Symptoms Scale
VDR	vitamin D receptor
BPH	benign prostatic hyperplasia
CV	coefficients of variation
DHEA	dehydroepiandrosterone
SHBG	sex hormone binding globulin
BMI	body mass index
PASE	Physical Activity Scale of the Elderly
FFQ	food frequency questionnaire
GLM	General Linear Model
