# Supplementary material for: Association of Circulating 25(OH)D and Lower Urinary Tract Symptoms: A Four-Year Prospective Study among Elderly Chinese Men
Source: Nutrients. 2016 May 7;8(5):273. doi: 10.3390/nu8050273 (PMC4882686; doi:10.3390/nu8050273)
Supplement: Supplementary file 1 [file nutrients-08-00273-s001.docx]

Supplementary Materials: Association of Circulating 25(OH)D and Lower Urinary Tract Symptoms: A 4-Year Prospective Study among Elderly Chinese Men

Zhao-Min Liu, Carmen Ka Man Wong, Dicken Chan, Jean Woo, Yu-Ming Chen, Bailing Chen, Lap-AhTse and Samuel Yeung-Shan Wong

**Table S1.** Results of univariate analysis between serum 25(OH)D level and International Prostate Symptoms Score (IPSS) change at 4-year follow-up by adjusting serum hormone levels in elderly men with baseline 25(OH)D ≤ 60 nmol/L (*n* = 196).

| **Serum Hormones** | **β** | ***p*** |
| --- | --- | --- |
| SHBG (nmol/L) | 0.117 | 0.099 |
| Bioavailable estradiol (pmol/L) | 0.012 | 0.862 |
| Total testerosterone (nmol/L) | 0 | 1.000 |
| Free testosterone (nmol/L) | −0.059 | 0.409 |
| Adrostenedione (ng/mL) | 0.166 | 0.019 |
| Dehydropiandrosterone (ng/mL) | 0.148 | 0.037 |
| 5-androstene-3b,17b-diol (ng/mL) | 0.112 | 0.115 |
| Dehydroepiandrosterone sulfate (ng/mL) | 0.049 | 0.495 |
| PTH (pmol/L) | −0.060 | 0.408 |
